# Characterization of the complete chloroplast genome of *Lonicera tatarica* L. (Caprifoliaceae)

**DOI:** 10.1080/23802359.2021.1934140

**Published:** 2021-06-28

**Authors:** Wangjun Yuan, Yuanyuan Ma, Sheqi He, Dongwen Chang, Yanxia He

**Affiliations:** a School of Pharmacy, Henan University, Kaifeng, China; b School of Life Sciences, Henan University, Kaifeng, China

**Keywords:** Chloroplast genome, *Lonicera tatarica*, phylogenetic tree

## Abstract

*Lonicera tatarica* L. is an excellent landscaping shrub with high ornamental value. Here, we report the complete chloroplast genome sequence of *L. tatarica*. The size of the chloroplast genome is 154,675 bp in length, including a large single copy region (LSC) of 88,361 bp, a small single copy region (SSC) of 18,750 bp, and a pair of inverted repeated regions of 23,782 bp. The *L. tatarica* chloroplast genome encodes 131 genes, including 85 protein-coding, 38 tRNA, and 8 rRNA genes. Phylogenetic analysis fully resolved *L. tatarica* in a clade with *L. japonica*, *L. confusa*, and *L. maximowiczii.* These data provide a useful resource when studying the genetic diversity of *L. tatarica.*

The genus *Lonicera* is classified in Caprifoliaceae and comprises more than 200 species (Naugžemys et al. 2007). Some of the taxa assigned to in this genus are used to facilitate erosion control as well as for ornamental purposes. One of them, *Lonicera tatarica* L., is an excellent landscaping shrub with high ornamental value due to its strong resistance, its ability to easily propagate, and beautiful flowers and leaves (Palacios et al. 2002). The emergence of high-throughput sequencing technologies makes it possible to quickly obtain a large amount of genomic data (He et al. 2017). Using this technology, this study reports the complete chloroplast (cp) genome of *L. tatarica*. The raw sequence data have been deposited in NCBI SRA with a project accession of PRJNA687839, and the cp genome sequence has been registered in GenBank with the Accession no. MW340876.

Fresh leaf samples of *L. tatarica* were collected from the campus of Henan University, China (34°49'19.76"N, 114°18'51.25"E), and a voucher specimen was deposited at the Herbarium of Henan University (Voucher no. HENU20200529, Kaifeng, Henan Province, China, Wangjun Yuan and 10200068@vip.henu.edu.cn). The total genomic DNA was extracted using a modified SDS method (Lim et al. 2016). The high-quality DNA was fragmented by sonication to a size of 350 bp. The paired-end libraries were sequenced using Illumina NovaSeq PE150 at Beijing Novogene Bioinformatics Technology Co., Ltd (Beijing, China). This yielded about 5G of raw data. Low-quality bases (mass value ≤20) over a certain percentage (the default was 40%) were removed, and some bases the overlap between them and the Adapter exceeding a certain threshold (the default was 15 bp) and less than 3 mismatches between them were thrown out. Approximately 2.5 Gb clean data were obtained, which were trimmed and assembled into the complete cp genome of *L. tatarica* using a CLC Genomics Workbench9.5.2 (CLC Inc., Rarhus, Denmark) with the sequence of *L. maackii* (GenBank Accession no. MN256451) as a reference. Then the cp genome was annotated using Geneious R11 (Biomatters, Auckland, New Zealand) while following the description provided by Liu et al. (2017). The phylogenetic position of *L. tatarica* was inferred using the whole cp genome sequences, and the maximum-likelihood (ML) method with nucleotide substitution model GTR + G and was implemented in RAxML-HPC v8.1.11 on the CIPRES cluster (Stamatakis 2014).

The complete cp genome of *L. tatarica* possesses a typical quadripartite structure with a length of 154,675 bp, which includes two inverted repeat (IR) regions of 23,782 bp, a large single-copy (LSC) region of 88,361 bp, and a small single-copy (SSC) region of 18,750 bp. The genome contains a total of 131 genes (85 protein-coding genes, 8 ribosomal RNA genes, and 38 tRNA genes). Among these genes, nine protein-coding genes (*atpF*, *ndhA*, *rpoc1*, *ndhB*, *petD*, *rpl16*, *rpl2*, *rps12*, and *rps16*) contain one intron, whereas two genes (*clpP* and *ycf3*) contain two introns. Notably, the *rps12* gene was trans-spliced with the 5′ end located in the LSC region and the 3′ end duplicated in the IR region. Additionally, the overall GC content was 38.4%, and the corresponding values of the LSC, SSC, and IR regions were 34.2%, 30.0%, and 42.5%, respectively. Furthermore, the phylogenic assessment revealed that *L. tatarica* was closely clustered with *L. japonica*, *L. confusa*, and *L. maximowiczii* with high bootstrap support values (Figure 1), and the topology of Caprifoliaceae, Valerianaceae and Dipsacaceae is identical to that of Xiang et al. (2020). These data provide a useful resource when studying the genetic diversity of *L. tatarica*.

**Figure 1.**
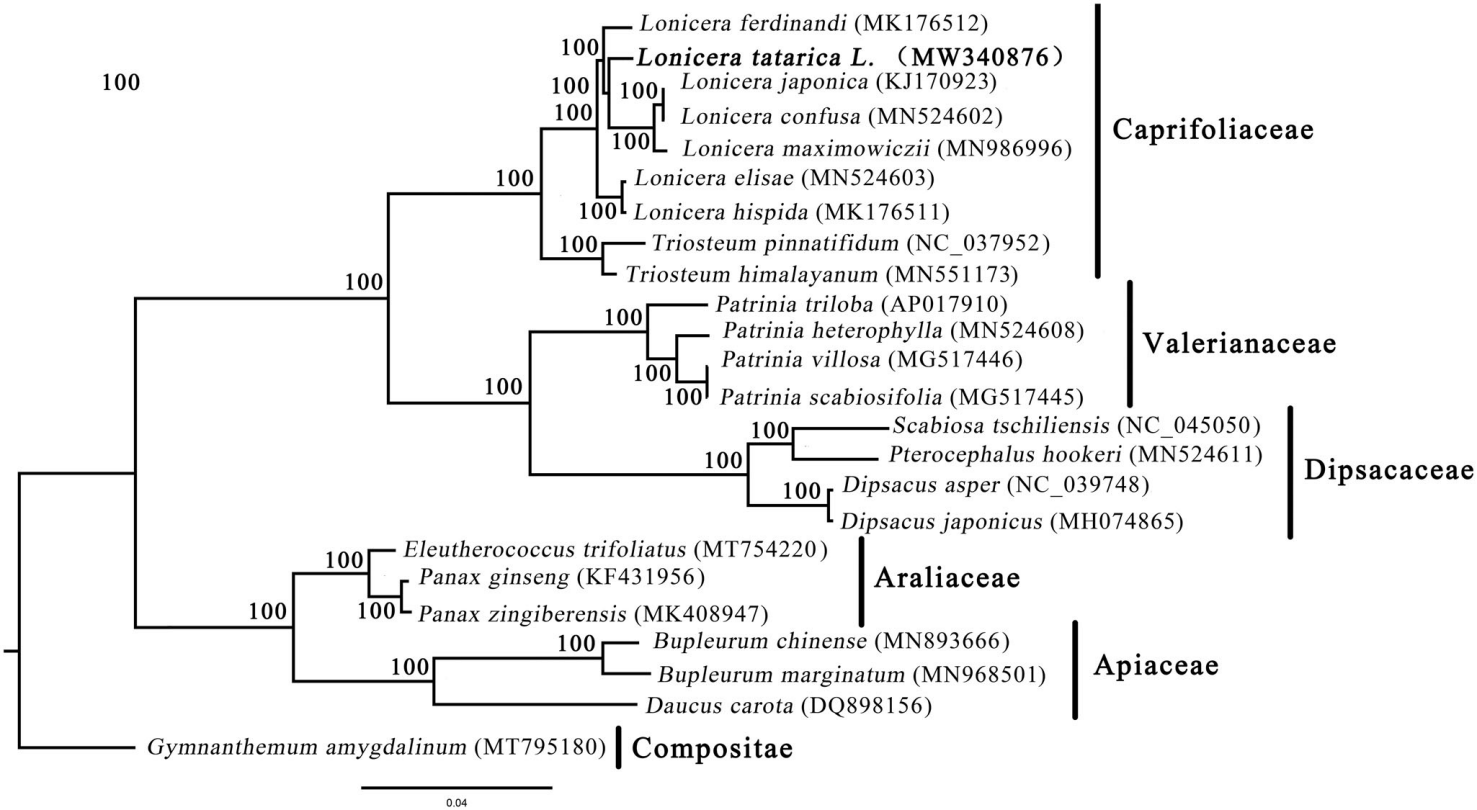
The phylogenetic tree based on 24 chloroplast genomes. Number above each node indicates the ML bootstrap support values.

## Data Availability

The genome sequence data that support the findings of this study are openly available in GenBank of NCBI at (https://www.ncbi.nlm.nih.gov/) under the Accession no. MW340876. The associated ﹡BioProject﹡, ﹡SRA﹡, and﹡Bio-Sample﹡ numbers of the raw sequence data and the genome are PRJNA687839, SRP300309, and SAMN17156276, respectively.
